# Thermoregulatory, cardiovascular, and metabolic responses to mild caloric restriction in the Brown Norway rat

**DOI:** 10.1002/phy2.16

**Published:** 2013-06-28

**Authors:** Cenk Aydin, Christopher J Gordon

**Affiliations:** 1 Department of Physiology, Faculty of Veterinary Medicine, University of UludagNilufer, Bursa, 16509, Turkey; 2 Toxicity Assessment Division, National Health and Environmental Effects Research Laboratory, Office of Research and Development, U.S. Environmental Protection AgencyResearch Triangle Park, North Carolina, 27711

**Keywords:** Caloric restriction, longevity, metabolic rate, radiotelemetry, thermoregulation

## Abstract

Caloric restriction (CR) has been demonstrated to prolong the life span of a variety of species. CR-induced reduction in core temperature (Tc) is considered a key mechanism responsible for prolonging life span in rodents; however, little is known about the regulation of CR-induced hypothermia as a function of the circadian cycle. We assessed how mild CR that resulted in a 10% reduction in body weight affected the 24 h patterns of Tc as well as heart rate (HR) and motor activity (MA) of the Brown Norway rat. Telemetered rats were allowed to feed for 20 weeks ad libitum (AL) or given a CR diet. Tc, HR, and MA of CR rats exhibited nocturnal reductions and diurnal elevations, opposite to that of AL rats. The effects of CR appeared to peak at ∼4 weeks. Metabolic rate (MR) and respiratory exchange ratio (RER) were measured overnight after 18 weeks of CR. MR and RER were elevated markedly at the time of feeding in CR rats and then declined during the night. We found that the pattern of Tc was altered with CR, characterized by elimination of high nocturnal Tc's typically observed in AL animals. In terms of mechanisms to prolong life span in CR animals, we suggest that the shift in the pattern of Tc during CR (i.e., elimination of high Tc's) may be as critical as the overall mean reduction in Tc. Future studies should address how the time of feeding may affect the thermoregulatory response in calorically restricted rats.

## Introduction

Since the initial report by McCay and Crowell (1934) showing that underfed rats lived longer than rats fed ad libitum (AL), many studies have been performed revealing that caloric restriction (CR) without malnutrition increased both the maximum and mean life spans of laboratory rodents. Aside from slowing aging, CR provides many health benefits. It delays or prevents many age-related diseases such as cardiovascular disease and cancer (Dirks and Leeuwenburgh 2006). CR involves a shift from state of growth and proliferation to maintenance and repair (Yu et al. 1985) and is most effective when caloric intake of animals fed AL is reduced by 20–40% without malnutrition (Anderson et al. 2009).

Energy homeostasis has emerged as an important regulator of longevity and aging. Energy balance depends on the mechanisms that regulate and coordinate food intake and the different components of energy expenditure, including metabolic requirements for thermoregulation and physical activity. When food is in short supply, caloric intake is lower than energy expenditure, resulting in a negative energy balance (Hill et al. 1985). Physiological responses to CR include depression in metabolic rate (MR) (Sohal and Weindruch 1996) and hypothermia (Rikke et al. 2003; Mayers et al. 2009; Swoap and Gutilla 2009). Moreover, there is an overall reduction in sympathetic activity to multiple regions, including brown adipose tissue (Young and Landsberg 1977), a reduction in heart rate (HR) and blood pressure (Young et al. 1978) but an increase in sympathetic activity to white adipose tissue (Migliorini et al. 1997). In the last few years, studies have shown that white adipose tissue is a very active endocrine gland which secretes a variety of peptides, cytokines, and complement factors. This is in addition to the presence of substrates, such as glycerol and free fatty acids, that are stored and released by fat cells and are known to have a major role in hepatic and peripheral glucose metabolism (Kershaw and Flier 2004).

The association between CR and hypothermia in rodents has spurred work toward a theory that a reduction in body temperature affords protection from damaging effects of oxidative stress, leading to significant longevity (Turturro and Hart 1991; Rikke et al. 2003; Tabarean et al. 2010). Simply stated, the reduction in metabolic heat production forces the rodent to regulate a lower core temperature (Tc). The reduced metabolism and Tc lessen the production of damaging free radicals, slowing essentially all metabolic processes associated with aging (Sohal and Weindruch 1996; Rikke et al. 2003). Of course, this overly simplified mechanism does not take into account the complex, circadian thermoregulatory response to CR. It is possible that the typical high body temperatures manifested during the night of a nocturnal animal may be moderated by CR. CR studies are often carried out in rodents fed in the daytime (Duffy et al. 1990b; Lambert and Merry 2004; Jang et al. 2012). Rats on a scheduled CR fed during the daytime undergo a shift in their circadian temperature rhythm, becoming hypothermic during the night and normothermic or hyperthermic in the daytime when they anticipate being fed (Gordon and Padnos 2002). It remains to be shown how the circadian control of metabolic thermogenesis and other autonomic responses such as HR respond in the face of CR with daytime feeding.

In addition to the magnitude of hypothermia, the change in temperature over the circadian cycle as well as the adaptation of the thermoregulatory system to continuous CR have not been addressed. Hence, this study sought to improve on the over simplified assumption that CR leads to hypothermia which contributes to increasing life span. We chose the Brown Norway rat because this strain is lean and does not become excessively obese when given an ad lib diet. The Brown Norway rat should provide a good model to study autonomic responses to CR over relatively long periods of time (Matsumoto et al. 2000). The major goal of this study was to assess how mild CR affects the patterns of regulation of Tc, HR, motor activity (MA), MR, and respiratory exchange ratio (RER) in telemetered Brown Norway rats. Moreover, we sought to quantify the time course of physiological and behavioral adaptation to CR.

## Materials and Methods

### Animals

Eleven male Brown Norway rats (Charles River Laboratories, Raleigh, NC) were obtained at 110 days of age and housed individually in acrylic cages lined with wood chip bedding at an ambient temperature of 22°C, 50% relative humidity, and a 12 h–12 h light–dark photoperiod (lights on at 06:00 am). Food (LabDietR manufactured by PMI Nutrition International, St. Louis, MO) and water were provided AL to all rats prior to CR. All surgical and testing procedures were approved by the USEPA Institutional Animal Care and Use Committee.

### Surgery

At an age of ∼120 days, rats were anesthetized with isoflurane in 100% oxygen (4.5% initially followed with 2% to maintain a surgical plane). The abdominal area was shaved and prepared for aseptic surgery. A midline abdominal incision was made to implant a radio transmitter to monitor HR, Tc, and MA (model CTA-F40; Data Sciences International, St Paul, MN). The electrocardiogram (ECG) leads were tunneled under the skin and positioned to detect the ECG. The body of the transmitter was sutured to the wall of the abdomen and closed with 4-0 silk. The skin was closed with surgical staples, and rats were administered an analgesic (buprenorphine, 0.03 mg/kg, s.c.) twice per day for 48 h. Rats were allowed 10 days of recovery prior to handling or testing. For additional details on telemetry surgery, see Gordon (1994).

### Telemetry protocol

After collecting a week of baseline telemetry data, the rats were assigned to an AL (*N* = 6) or calorically restricted (*N* = 5) feeding regimen for 20 weeks. The CR group was fed their allotment of food at ∼11:00 am each day. The amount of food provided each day was calculated using a previously published algorithm designed to achieve a 10% reduction in body weight below that of the AL level (Ali et al. 1992). Briefly, this is achieved by initially feeding the CR animals a smaller portion of feed (∼10 g/day) that is then adjusted daily to maintain body weight that is 90% of that of the AL group (∼15 g/day to maintain weight). All rats were weighed daily at ∼07:00 am. The body weight data were used to determine the amount of food given to the CR group each day. It should be noted that for the young rats, the AL fed animals were continuing to grow and gain weight. The algorithm used the average weight of the AL group as a means of calculating the 90% weight for the CR group. Hence, the weight of the young CR group increased gradually, in a parallel fashion with the AL group for the 20-week treatment period.

### Calorimetry

The telemetered rats described above were housed individually in calorimeters following 18 weeks of CR (Oxymax, Columbus Inst., Columbus, OH) to measure oxygen consumption, carbon dioxide production, and RER. Rats were placed in individual calorimeter chambers made of clear plastic (length 30.4 cm, width 19.04 cm, height 19.04 cm) at 09:00 am and remained undisturbed for next 22 h. The calorimeters had perforated plastic floor to allow feces and urine to drop through. Fresh, dry air was pumped into each calorimeter at a controlled rate 2.0L/min. The calorimeter was housed in the animal vivarium maintained at 22°C. Telemetry boards were placed beneath the calorimeter to collect telemetry data. Ad lib rats were given a full food tray in the calorimeter. The CR rats received their food allotment at 11:00 am as this was their normal feeding time. Water was available ad lib in each group in the calorimeter. To feed the animals, the lids of the calorimeters had to be removed temporarily and then replaced, a process that required about 30 sec. At the same, the lids of the ad lib animals were also opened in a similar way to mimic the disturbance and stirring of the air in the calorimeter.

The Oxymax software provided by the manufacturer provides a measure of heat production in kcal/h and RER. Heat production was converted to MR in units of W/kg. The calorimeter was calibrated prior to the start of each experiment using a certified gas mixture standard of oxygen, carbon dioxide, and nitrogen. Telemetry data were monitored and recorded as explained previously (Gordon 1994).

### Statistics

Extraneous values of Tc, HR, and MA monitored by telemetry were first clipped using the analysis program provided by the manufacturer. (Data Science International, St Paul, MN). Any HR <200 or >600 beats/min and temperature data <34 or >41°C were removed. A “foldagram” averaging routine provided by the telemetry manufacturer was used to average telemetry data over a 1 week period ensemble into a 24-h period. These data were analyzed using repeated measures analysis of variance (RMANOVA) using treatment (CR vs. AL) and time as a repeated factor (Sigma Plot, Version 11.0, Point Richmond, CA). The variances of data were analyzed using the Shapiro–Wilk test for normality of variances. Daily body weight data were also evaluated using RMANOVA. Data from the calorimeter were averaged into 1 h bins and analyzed with RMANOVA.

## Results

### Body weight

**Figure 1:**
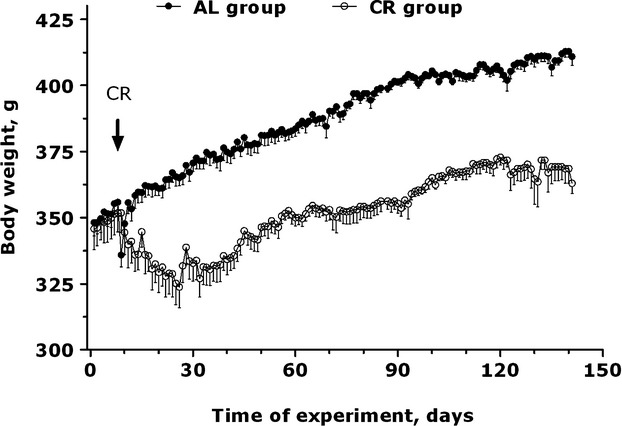
Time course of daily body weight for ad libitum (AL) and caloric restricted (CR) rats. Data plotted as mean ± SE (*N* = 6 for AL; *N* = 5 for CR). RMANOVA: treatment, *F* (1, 9) = 81.54, *P* < 0.001; day, *F* (154, 1386) = 63.42, *P* < 0.001; treatment × day, *F* (154, 1386) = 7.38, *P* < 0.001.

There was no difference in body weight between the AL and CR groups at 4 months of age prior to the start of the food restriction protocol. At the onset of CR, body weight decreased from 350 to 325 g over a period of 20 days (Fig. 1). Body weight was stable at this time and then increased in a parallel fashion to that of the AL group. Body weight of the control group increased from 350 to 420 g over a period of 140 days. Overall, during the period of CR, body weight of the restricted group was 10% below that of the AL group.

### Telemetry

**Figure 2:**
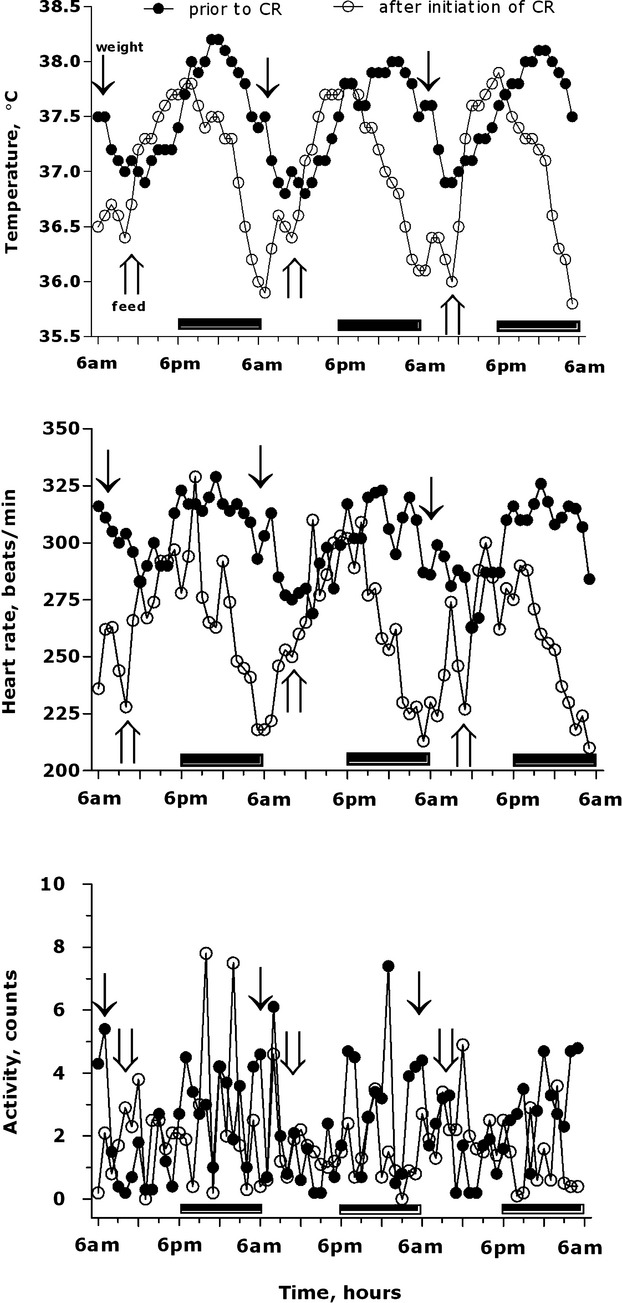
Example of telemetric recordings of core temperature, heart rate, and motor activity of a single rat prior to CR and on days 4–6 of CR. Small arrows indicate time of daily weighing at 07:00 am; large arrows indicate time of feeding at 11:00 am where rat is receiving ∼10 g of food per day.

A representative plot for a single rat before and after 3 days of CR demonstrates the dynamic, circadian responses of Tc, HR, and MA (Fig. 2). CR led to marked changes after just 3 days that were clearly influenced by the circadian cycle. Elevations in Tc, HR, and activity occur with the determination of body weight. At the onset of the dark phase, a reduction in Tc, HR, and MA are apparent; these parameters remain depressed until the next determination of body weight.

**Figure 3:**
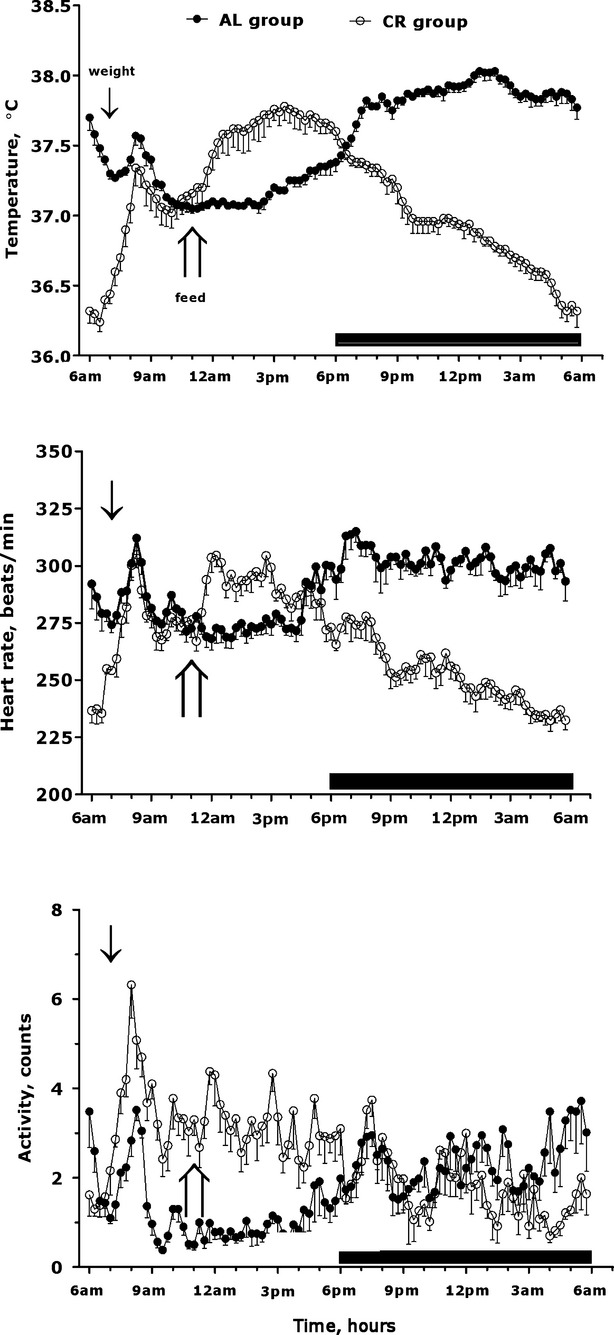
Foldagrams for core temperature, heart rate, and motor activity of AL and CR groups during the fourth week of caloric restriction. Foldagrams represent 7 days of data averaged into 1 day for each rat. Data plotted as mean ± SE. Arrow symbols denote weighing and feeding (see Fig. 2).

Foldagram analysis of the telemetry variables after 4 weeks of CR illustrates the advance phase shift of Tc, HR, and MA during the light and dark cycles of the AL and CR groups (Fig. 3). This type of analysis eliminated most of the noise from the ultradian rhythms and the variation from individual animals. At 06:00 am Tc, HR and MA of the AL rats decreased from their elevated nocturnal levels while that of the CR group increased rapidly. There is a slight but transient increase in the AL group when the animals are weighed at 07:00 am. HR and Tc of the CR animals also rose transiently whereas MA increased markedly and remains elevated throughout the pre- and postfeed periods. We consider these elevations to be attributed to a combination of handling and anticipation of the weighing procedure. Upon feeding, HR and Tc increased and remained elevated for several hours and then exhibited gradual reductions throughout the remainder of the light phase and throughout the dark phase (Fig. 3). MA during week 4 of the AL and CR groups was similar through most of the dark phase with that of the CR group decreasing to minimal levels at ∼05:00 am.

**Figure 4:**
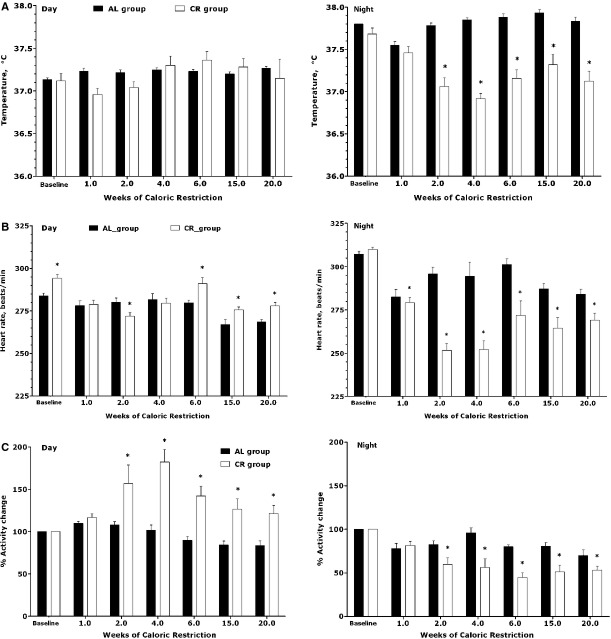
(A) Time course of day and night time core temperature (A), over the course of 20 weeks of CR. Asterisks indicate significant difference when compared to ad lib fed animals compared at each time point. RMANOVA: Day: treatment, *F* (1, 9) = 0.0094, *P* < 0.92; week, *F* (7, 63) = 2.77, *P* < 0.014; treatment × week, *F* (7, 63) = 1.869, *P* < 0.090. Night: treatment, *F* (1, 9) = 32.83, *P* < 0.001; week, *F* (7, 63) = 5.56; treatment × week, *F* (7, 63) = 8.07, *P* < 0.001. (B) Time course of day and night time heart rate (B) over the course of 20 weeks of CR. Asterisks indicate significant difference when compared to ad lib fed animals compared at each time point. RMANOVA: Day: treatment, *F*(1,9) = 12.81, *P* = 0.006; week, *F* (7, 63) = 17.81, *P* < 0.001; treatment × week, *F* (7, 63) = 4.48, *P* < 0.001. Night: treatment, *F* (1, 9) = 34.62, *P* < 0.001; week, *F* (7, 63) = 16.95, *P* < 0.001; treatment × week, *F* (7, 63) = 10.61, *P* < 0.001. (C) Time course of day and night time motor activity (C) over the course of 20 weeks of CR. Asterisks indicate significant difference when compared to ad lib fed animals compared at each time point. RMANOVA: Day: treatment, *F* (1, 9) = 21.60, *P* < 0.001; week, *F* (7, 63) = 16.97, *P* < 0.001; treatment × week, *F* (7, 63) = 8.42, *P* < 0.001. Night: treatment, *F* (1, 9) = 22.76, *P* < 0.001; week, *F* (7, 63) = 12.94, *P* < 0.001; treatment × week, *F* (7, 63) = 5.14, *P* < 0.001.

Foldagram analysis of telemetry data during weeks 1, 2, 4, 6, 15, and 20 CR indicated marked light versus dark cycle effects (Fig. 4A–C). Daytime Tc (averaged from 6 am–6 pm) was unaffected by CR when it is averaged over the entire light phase (6 am–6 pm). HR during the light phase was also minimally affected by CR whereas MA was markedly elevated in the CR group. Nocturnal Tc and HR were significantly reduced, an effect that reached a maximal effect after 4 weeks of CR (Fig. 4A and B). MA expressed as percentage of the activity level recorded during the baseline period, reached a nadir after 6 weeks of CR and then rose slightly by week 20 (Fig. 4C).

### Calorimetery

**Figure 5:**
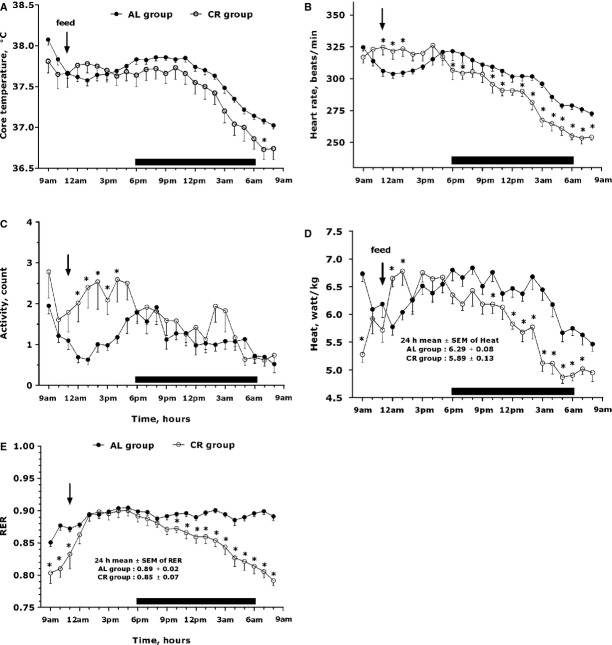
(A) Time course of telemetry data (A–C) in the AL and CR housed in the calorimeter for 22 h (after 6 weeks of caloric restriction). Asterisks indicate significant difference when compared to ad lib fed animals compared at each time point. RMANOVA: Core Temperature: treatment, *F* (1, 9) = 1.09, *P* < 0.322; week, *F* (23, 207) = 96.35, *P* < 0.001; treatment × week, *F* (23, 207) = 5.76, *P* < 0.001. Heart Rate: treatment, *F* (1, 9) = 4.04, *P* < 0.075; week, *F* (23, 207) = 41.40, *P* < 0.001; treatment × week, *F* (23, 207) = 6.13, *P* < 0.001. Activity: treatment, *F* (1, 9) = 1.99, *P* < 0.191; week, *F* (23, 207) = 6.77, *P* < 0.001; treatment × week, *F* (23, 207) = 3.09, *P* < 0.001. (B) Time course of calorimetery measurements including metabolic rate (D) and respiratory exchange ratio (RER) (E) in the AL and CR housed in the calorimeter for 22 h (after 18 weeks of caloric restriction). Asterisks indicate significant difference when compared to ad lib fed animals compared at each time point. RMANOVA: Core Temperature: treatment, *F* (1, 9) = 1.09, *P* < 0.322; week, *F* (23, 207) = 96.35, *P* < 0.001; treatment × week, *F* (23, 207) = 5.76, *P* < 0.001. Heart Rate: treatment, *F* (1, 9) = 4.04, *P* < 0.075; week, *F* (23, 207) = 41.40, *P* < 0.001; treatment × week, *F* (23, 207) = 6.13, *P* < 0.001. Activity: treatment, *F* (1, 9) = 1.99, *P* < 0.191; week, *F* (23, 207) = 6.77, *P* < 0.001; treatment × week, *F* (23, 207) = 3.09, *P* < 0.001.

When transferred to the calorimeter, HR, Tc, and MA were all elevated above the levels typically seen in the home cage (Fig. 5A–C). This is presumably a stress response to a novel environment. After being housed over night in the calorimeter, the telemetry variables were all reduced to levels seen in their home cages. Significant elevations in HR were observed during the light phase, including the pre- and postfeed periods. HR was significantly reduced during onset of the dark period and persisted throughout the next day. MA was also elevated throughout the light phase and was equal to the AL levels during the night. Tc decreased through the dark cycle but was significantly below that of the AL animals the following day.

MR was reduced significantly in CR rats when first placed in the calorimeter (Fig. 5D). RER was also significantly reduced in the restricted group in the morning (Fig. 5E). When given their food allotment, MR and RER increased in the CR group; RER was the same in both groups throughout the rest of the light phase and first few hours of darkness. MR increased significantly when the CR rats were fed, then returned to that of the AL group and then declined gradually below the AL group during the latter part of the dark phase and through the morning hours until the rats were removed from the calorimeter. The overall mean values over the 22-h period in AL group and CR group were 6.29 ± 0.08 and 5.89 ± 0.13 W/kg for MR and 0.89 ± 0.02 and 0.85 ± 0.07 W/kg for RER, respectively.

## Discussion

**Figure 6:**
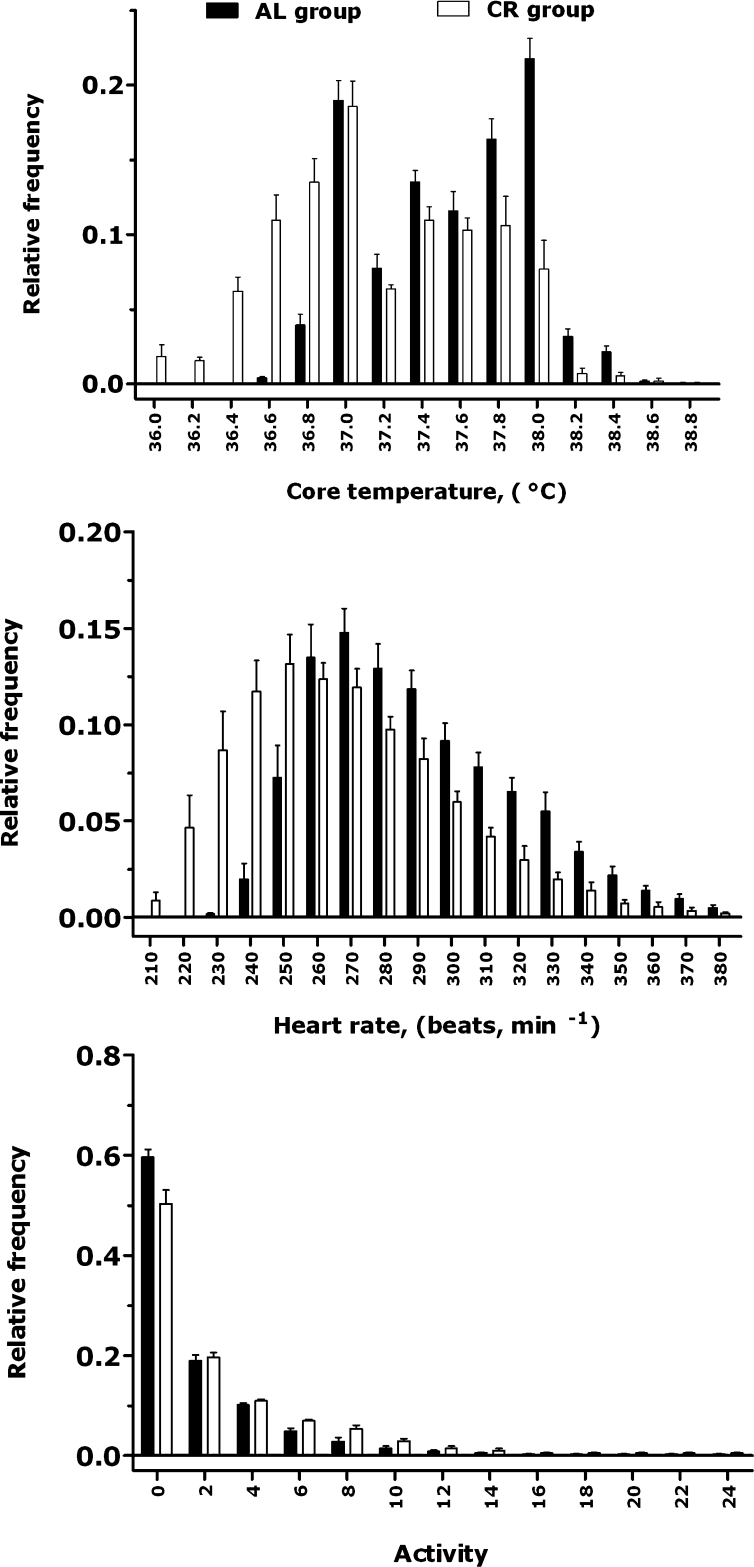
Frequency distribution of core temperature, heart rate, and motor activity during the fourth week of caloric restriction. Data plotted as mean ± SE. *N* = 6 for AL and *N* = 5 for CR.

We have shown that a relatively mild level of CR resulting in a 10% reduction in the ad lib body weight led to significant reductions in Tc, HR, MR, RER, and elevation in MA in the Brown Norway rat. Application of radiotelemetry in unstressed and undisturbed animals showed that rats respond quickly to CR (∼24 h) and show peak physiological responses after ∼4 weeks of restriction. In addition, the circadian physiological rhythms adapt rapidly to a diurnal CR paradigm. That is, when calorically restricted rats are given their food allotment in middle of the light period, they undergo a phase shift in the circadian pattern of their thermoregulatory and cardiovascular processes that is apparently geared to anticipate both the daytime feeding and lack of food availability at night. Within a few days after the start of CR, the rats exhibited an elevated level of MA during the day and a marked reduction in Tc, HR, and MR at night and in the early morning hours prior to feeding. While the 12 h averages of Tc during the daytime are unaffected by CR, there is nonetheless a smaller but significant rise in temperature for several hours following daytime feeding. Finally, the frequency distribution analysis of Tc illustrates how CR leads to a shift from bimodal to unimodal distribution, with elimination of the high Tcs normally exhibited at night (Fig. 6). We contend that this type of analysis may improve our understanding of the mechanisms of action of the life extension efficacy of CR.

The calorimeter experiments demonstrate that the calorically restricted Brown Norway rats undergo a nightly reduction in MR and RER. This corroborates other rodents' studies showing that CR leads to a metabolic savings and predominant metabolism of lipids when not feeding (Bevilacqua et al. 2005; Bruss et al. 2010). The telemetry monitoring while animals are housed in the calorimeter suggests that initial placement in the calorimeter results in a stress-induced rise in Tc and HR. This stress from placement in the novel calorimeter environment is unavoidable. Nonetheless, once the animals settled and were given their food allotment, a clear rise in MR and RER were evident during feeding. These data suggest that, while CR eventually leads to metabolic savings late in the nocturnal phase, there is a metabolic cost associated with daily feeding compared with that of the AL animals. On the other hand, the hypothermic effect of CR may have a crucial impact on metabolic savings (see below). The calorimeter experiment also corroborated the MA data collected in the home cage studies showing that CR leads to an increase in daytime activity and little change in activity at night.

Physical activity represents a major part of an endotherm's energy budget. Under CR one would anticipate that animals would reduce their levels of activity to conserve energy. Paradoxically, rodents subject to CR undergo an increase in levels of MA (Duffy et al. 1990a). Indeed, CR in this study led to an overall increase in MA over the 24-h period with the principal rise in activity occurring during the daylight hours. For example, after 4 weeks of CR, daytime MA increased by 263%, night time MA decreased by just 33%; overall. Twenty-four hour MA increased by 141% in the CR group. On the other hand, the calorimetry study suggests that there is an overall energy savings of ∼7% in CR animals in spite of an elevated level of MA. We suspect that this is achieved by the CR-induced reduction in Tc via a Q_10_-mediated reduction in metabolic processes. It will be of interest to understand how animals of a CR regimen may utilize thermoregulation to achieve significant energy savings while simultaneously exhibiting a state of hyperactivity.

HR of the CR rats was above that of the AL group for just a few hours after feeding (cf. Fig. 4). Otherwise, HR remained below that of the AL levels, reaching a minimum 30% reduction at the onset of the dark to light phase after 4 weeks of CR. Knight et al. (2011) observed in male FBNF1 rats a remarkable stability of CR-induced bradycardia for an entire year of CR regardless of ambient temperature. Spectral analysis of HR in male Sprague-Dawley rats suggests that CR-induced bradycardia may be due to increased vagal tone (Mager et al. 2006). It is interesting to note the lack of correlation between MA and HR in the CR rats over the 24-h period such as graphed in Figure 3. In telemetered rodents, HR and MA are positively correlated with a relatively high correlation coefficient (Meinrath and D'Amato 1979); however, we find that the CR rat exhibits a profound reduction in HR at night while MA was reduced slightly but statistically indifferent from that of the AL group. Overall, HR is controlled by a balance between sympathetic–parasympathetic pathways along with direct influences of Tc (i.e., Q_10_ effect) on the pacemaker. This latter component should play a greater role in the CR rodent that sustains significant reductions in Tc. It will be of interest to understand these relationships in the regulation of HR in CR rats and other species.

We suggest that calculation of the frequency distribution of Tc may represent a valuable method to understand how the thermoregulatory response to CR may extend life span (Fig. 6). The unimodal distribution of HR is simply shifted to lower values with CR. The distribution of MA is essentially unaffected by CR (Fig. 6). On the other hand, the distribution of Tc of the AL group was bimodal, reflecting the periodicity of low, day time and high, night time Tcs. However, the nocturnal peak exhibited by AL fed animals is nearly abolished with CR. This means that CR rats are not subjected to the night exposures to high temperatures normally encountered with AL feeding. As an example, the percentage of Tcs in AL animals over a 24-h period that was ≥38°C is 27.6%; this value drops to 9.3% with CR. Contrarily, 52.7% of the temperatures of caloric restricted rats are manifested at Tc ≤37.0°C, whereas this value drops to 23.3% in AL fed animals. We suggest that the frequency distribution of Tc allows one to develop a temperature-temporal spectrum over the life of a CR versus an AL fed rat. In other words, the hypometabolic and hypothermic effects of CR are generally considered to be protective and afford an increase in life span. Peak, nocturnal rises in body temperatures over the life of an animal may represent a toll on cellular processes that is markedly attenuated during CR.

## References

[b1] Ali JS, Olszyk VB, Lee KLA, Kendall SM, Rhoderick RR, Bushnell PJ (1992). A lotus 1-2-3-based system for recording and maintaining body weight of laboratory animals. Behav. Res. Methods Instrum. Comput.

[b2] Anderson RM, Shanmuganayagam D, Weindruch R (2009). Caloric restriction and aging: studies in mice and monkeys. Toxicol. Pathol.

[b3] Bevilacqua L, Ramsey JJ, Hagopian K, Weindruch R, Harper ME (2005). Long-term caloric restriction increases UCP3 content but decreases proton leak and reactive oxygen species production in rat skeletal muscle mitochondria. Am. J. Physiol. Endocrinol. Metab.

[b4] Bruss MD, Khambatta CF, Ruby MA, Aggarwal I, Hellerstein MK (2010). Calorie restriction increases fatty acid synthesis and whole body fat oxidation rates. Am. J. Physiol. Endocrinol. Metab.

[b5] Dirks AJ, Leeuwenburgh C (2006). Caloric restriction in humans: potential pitfalls and health concerns. Mech. Ageing Dev.

[b6] Duffy PH, Feuers R, Nakamura KD, Leakey J, Hart RW (1990a). Effect of chronic caloric restriction on the synchronization of various physiological measures in old female Fischer 344 rats. Chronobiol. Int.

[b7] Duffy PH, Feuers RJ, Hart RW (1990b). Effect of chronic caloric restriction on the circadian regulation of physiological and behavioral variables in old male B6C3F1 mice. Chronobiol. Int.

[b8] Gordon CJ (1994). 24-hour control of body temperature in the rat: II. Diisopropyl fluorophosphate-induced hypothermia and hyperthermia. Pharmacol. Biochem. Behav.

[b9] Gordon CJ, Padnos B (2002). Dietary exposure to chlorpyrifos alters core temperature in the rat. Toxicology.

[b10] Hill JO, Latiff A, DiGirolamo M (1985). Effects of variable caloric restriction on utilization of ingested energy in rats. Am. J. Physiol. Regul. Integr. Comp. Physiol.

[b11] Jang H, Lee G, Kong J, Choi G, Park YJ, Kim JB (2012). Feeding period restriction alters the expression of peripheral circadian rhythm genes without changing body weight in mice. PLoS ONE.

[b12] Kershaw EE, Flier JS (2004). Adipose tissue as an endocrine organ. J. Clin. Endocrinol. Metab.

[b13] Knight WD, Witte MM, Parsons AD, Gierach M, Overton JM (2011). Long-term caloric restriction reduces metabolic rate and heart rate under cool and thermoneutral conditions in FBNF1 rats. Mech. Ageing Dev.

[b14] Lambert AJ, Merry BJ (2004). Effect of caloric restriction on mitochondrial reactive oxygen species production and bioenergetics: reversal by insulin. Am. J. Physiol. Regul. Integr. Comp. Physiol.

[b15] Mager DE, Wan R, Brown M, Cheng A, Wareski P, Abernethy DR (2006). Caloric restriction and intermittent fasting alter spectral measures of heart rate and blood pressure variability in rats. FASEB J.

[b16] Matsumoto AM, Marck BT, Gruenewald DA, Wolden-Hanson T, Naai MA (2000). Aging and the neuroendocrine regulation of reproduction and body weight. Exp. Gerontol.

[b17] Mayers JR, Iliff BW, Swoap SJ (2009). Resveratrol treatment in mice does not elicit the bradycardia and hypothermia associated with calorie restriction. FASEB J.

[b18] McCay CM, Crowell MF (1934). Prolonging the life span. Sci. Monthly.

[b19] Meinrath M, D'Amato MR (1979). Interrelationships among heart rate, activity, and body temperature in the rat. Physiol. Behav.

[b20] Migliorini RH, Garofalo MA, Kettelhut IC (1997). Increased sympathetic activity in rat white adipose tissue during prolonged fasting. Am. J. Physiol.

[b21] Rikke BA, Yerg JE, Battaglia ME, Nagy TR, Allison DB, Johnson TE (2003). Strain variation in the response of body temperature to dietary restriction. Mech. Ageing Dev.

[b22] Sohal RS, Weindruch R (1996). Oxidative stress, caloric restriction, and aging. Science.

[b23] Swoap SJ, Gutilla MJ (2009). Cardiovascular changes during daily torpor in the laboratory mouse. Am. J. Physiol. Regul. Integr. Comp. Physiol.

[b24] Tabarean I, Morrison B, Marcondes MC, Bartfai T, Conti B (2010). Hypothalamic and dietary control of temperature-mediated longevity. Ageing Res. Rev.

[b25] Turturro A, Hart RW (1991). Longevity-assurance mechanisms and caloric restriction. Ann. N. Y. Acad. Sci.

[b26] Young JB, Landsberg L (1977). Suppression of sympathetic nervous system during fasting. Science.

[b27] Young JB, Mullen D, Landsberg L (1978). Caloric restriction lowers blood pressure in the spontaneously hypertensive rat. Metabolism.

[b28] Yu BP, Masoro EJ, McMahan CA (1985). Nutritional influences on aging of Fischer 344 rats: I. Physical, metabolic, and longevity characteristics. J. Gerontol.

